# A multicenter randomized phase 4 trial comparing sodium picosulphate plus magnesium citrate vs. polyethylene glycol plus ascorbic acid for bowel preparation before colonoscopy. The PRECOL trial

**DOI:** 10.3389/fmed.2022.1013804

**Published:** 2022-12-08

**Authors:** Valentina D’Angelo, Maria Carmela Piccirillo, Massimo Di Maio, Ciro Gallo, Cristina Bucci, Corrado Civiletti, Elena Di Girolamo, Pietro Marone, Giovanni Battista Rossi, Alfonso Mario Tempesta, Maura C. Tracey, Marco Romano, Agnese Miranda, Domenico Taranto, Gabriella Sessa, Pasquale Esposito, Raffaele Salerno, Rossella Pumpo, Francesca Romana De Filippo, Elisabetta Della Valle, Mario de Bellis, Francesco Perrone

**Affiliations:** ^1^Division of Gastroenterology and Digestive Endoscopy, Department of Abdominal Oncology, Istituto Nazionale Tumori–IRCCS–Fondazione G. Pascale, Napoli, Italy; ^2^Clinical Trial Unit, Department of Translational Research, Istituto Nazionale Tumori–IRCCS–Fondazione G. Pascale, Napoli, Italy; ^3^Department of Oncology, Ospedale Mauriziano, University of Turin, Torino, Italy; ^4^Medical Statistics Unit, University of Campania Luigi Vanvitelli, Napoli, Italy; ^5^Unit for Rehabilitation Medicine, Department for the Support of Oncological Patients Pathways, Clinical Activities and Critical Area, Istituto Nazionale Tumori–IRCCS Fondazione G. Pascale, Napoli, Italy; ^6^Division of Gastroenterology, University of Campania Luigi Vanvitelli, Napoli, Italy; ^7^Division of Gastroenterology, Clinica Mediterranea, Napoli, Italy; ^8^Division of Gastroenterology, ASST Fatebenefratelli Sacco, Milano, Italy; ^9^Digestive Endoscopy Unit, Ospedale S. Maria del Loreto Nuovo, Napoli, Italy; ^10^Division of Gastroenterology, Ospedale Riuniti San Giovanni di Dio e Ruggi D’Aragona, Salerno, Italy; ^11^Department of Public Health, University of Napoli Federico II, Napoli, Italy

**Keywords:** bowel preparation, sodium picosulphate plus magnesium citrate, polyethylene glycol plus ascorbic acid, patients compliance, colonoscopy, randomized controlled trial

## Abstract

**Background:**

Adequate bowel preparation before colonoscopy is crucial. Unfortunately, 25% of colonoscopies have inadequate bowel cleansing. From a patient perspective, bowel preparation is the main obstacle to colonoscopy. Several low-volume bowel preparations have been formulated to provide more tolerable purgative solutions without loss of efficacy.

**Objectives:**

Investigate efficacy, safety, and tolerability of Sodium Picosulphate plus Magnesium Citrate (SPMC) vs. Polyethylene Glycol plus Ascorbic Acid (PEG-ASC) solutions in patients undergoing diagnostic colonoscopy.

**Materials and methods:**

In this phase 4, randomized, multicenter, two-arm trial, adult outpatients received either SPMC or PEG-ASC for bowel preparation before colonoscopy. The primary aims were quality of bowel cleansing (primary endpoint scored according to Boston Bowel Preparation Scale) and patient acceptance (measured with six visual analogue scales). The study was open for treatment assignment and blinded for primary endpoint assessment. This was done independently with videotaped colonoscopies reviewed by two endoscopists unaware of study arms. A sample size of 525 patients was calculated to recognize a difference of 10% in the proportion of successes between the arms with a two-sided alpha error of 0.05 and 90% statistical power.

**Results:**

Overall 550 subjects (279 assigned to PEG-ASC and 271 assigned to SPMC) represented the analysis population. There was no statistically significant difference in success rate according to BBPS: 94.4% with PEG-ASC and 95.7% with SPMC (*P* = 0.49). Acceptance and willing to repeat colonoscopy were significantly better for SPMC with all the scales. Compliance was less than full in 6.6 and 9.9% of cases with PEG-ASC and SPMC, respectively (*P* = 0.17). Nausea and meteorism were significantly more bothersome with PEG-ASC than SPMC. There were no serious adverse events in either group.

**Conclusion:**

SPMC and PEG-ASC are not different in terms of efficacy, but SPMC is better tolerated than PEG-ASC. SPMC could be an alternative to low-volume PEG based purgative solutions for bowel preparation.

**Clinical trial registration:**

[ClinicalTrials.gov], Identifier [NCT01649674 and EudraCT 2011–000587–10].

## Introduction

The goal of colonoscopy should be safe, accurate, and complete examination of the entire colon. Therefore, colon cleansing before colonoscopy is crucial. Unfortunately, up to 25% of all colonoscopies have an inadequate bowel cleansing, which is due to different factors ranging from patient-related variables (compliance with preparation and/or medical conditions) to scheduling of colonoscopy (waiting times, timing of the exam, etc.) (1–3).

The ideal preparation for colonoscopy should clean the colon, with no alteration of colonic mucosa and without causing patient discomfort or shifts in fluids, as well as electrolytes (1–4). High quality bowel cleansing is obtained using high-volume (4 L) solutions of Polyethylene Glycol with electrolytes (PEG-ELS) (1, 2). However, 15% of patients do not complete the bowel preparation with this solution because of its large volume (4). Low-volume (2 L) PEG-ELS solutions were formulated to provide a more tolerable bowel preparation with similar efficacy (5–7). Nevertheless, still 5–10% patients do not assume the entire purgative solution (5–8). Sodium Mono Phosphate (NaP), a very low-volume (500 ml), osmotically active agent, was used to improve patient compliance for bowel preparation, but its use is not recommended because of risk for serious adverse events (8, 9). An alternative to NaP is Sodium Picosulfate and Magnesium Citrate (SPMC), another very low-volume (300 ml) bowel preparation (10). Previous studies comparing SPMC with low-volume PEG-ELS solutions demonstrated the non-inferiority of SPMC in bowel cleansing and suggested that SPMC is more tolerable than PEG-based solutions (11–20), even if one study showed that patient overall tolerance was higher for PEG-ASC than SPMC (12). However, the majority of these studies had a single-center design and evaluated a relatively small number of patients (10–16), with the exception of the two SEE CLEAR studies (19, 20).

This paper reports the results of a large, phase 4, randomized, multicenter study that investigated efficacy, safety, and tolerability of SPMC vs. PEG-ELS plus ascorbic acid (PEG-ASC) solutions in patients undergoing colonoscopy.

## Materials and methods

This non-profit multicenter, phase 4, randomized, two-arm trial compared SPMC versus PEG-ASC, in patients undergoing colonoscopy. The primary aim was to compare the quality of bowel preparation. The co-primary aim was to evaluate patient acceptance of the purgative solutions. The secondary aim was to compare patient compliance for bowel preparation. The study protocol was approved by the Ethics Committee of the coordinating center (March 29, 2011), and by the Ethics Committees of the other participating centers. The study was conducted in accordance with the Principles of the Declaration of Helsinki, Good Clinical Practice, and applicable regulatory requirements. All participants provided written informed consent at the time of the enrollment and randomization, which were coincident with the booking of colonoscopy. The trial was registered at ClinicalTrials.gov (NCT01649674) and EudraCT (2011—000587—10).

The study was open for the treatment assignment (both the endoscopist and the patient were aware of the type of preparation), but it was blind for the independent assessment of the primary end-point (reviewers were unaware of the bowel preparation assumed by patients). Patients were registered and randomized *via* a web platform^1^ at the Clinical Trial Unit of the coordinating center which collected all the data. Randomization was performed by using a minimization procedure that included center, indication for colonoscopy (screening vs. previous clinical or radiological suspicion of cancer vs. other), previous colonoscopy (no vs. yes), and modality of assumption (standard vs. split), as stratification variables. Sample size was calculated with the aim of recognizing a difference of 10% (considered as a clinically relevant minimum value) in the proportion of successes (adequate bowel preparation) between the two purgative solutions. Considering a success rate in the less effective arm equal to 80%, and a two-sided alpha error of 0.05, the study was going to guarantee 90% power in highlighting the expected difference of 10%, with the enrollment of 525 patients. With this sample size, and the same alpha error, the study has more than 99% power to recognize a difference of 1 point on the patients’ acceptance scale.

Patients, aged 18 years or older, were eligible if they were scheduled for a colonoscopy due to screening, diagnosis and follow-up after polypectomy. Subjects who had previously undergone bowel resection or had severe inflammatory bowel disease, renal failure, heart failure (NYHA classes III and IV), hepatic insufficiency (class B or C, according to the Child-Pugh classification), severe dehydration, as well as, pregnant women, individuals assuming lithium, and subjects with other known contraindications, either to bowel preparation (i.e., hypermagnesemia, rhabdomyolysis) or to execution of colonoscopy, were excluded.

The purgative solutions used for bowel preparation were respectively PEG-ASC and SPMC. Both bowel preparations were used in clinical practice at the time of the study. PEG-ASC (Moviprep, Norgine, Harefield, UK) is composed of PEG-3350, sodium sulfate, sodium chloride and ascorbic acid (2, 21). Ascorbic acid cannot be absorbed and functions as osmotic laxative, reducing the volume of the purgative solution to 2 L (4, 22). However, an additional volume of 1 L of clear fluid is required to reduce the risk of dehydration (4). SPMC (Citrafleet, Casen Recordati S. L., Zaragoza, Spain) is composed of sodium picosulfate and magnesium citrate which act as stimulant laxative, and osmotic laxative, respectively (2, 23). Despite the very low-volume (300 ml) of purgative solution, there is a need of an additional 3 L of clear liquids to avoid dehydration (4). Bowel preparation in both arms was done according to two different assumption modalities: standard (purgative solution was assumed entirely the evening before colonoscopy) or split (purgative solution was divided between the evening before and the morning of the colonoscopy). The modality of bowel preparation (standard or split) was declared before randomization and was considered among the stratification variables in the minimization procedure. Bowel preparation in the PEG-ASC - standard assumption arm consisted of 1 L of purgative solution followed by 0.5 L of clear liquids assumed twice, over a period of 90 min, respectively from 5:00 to 6:30 pm and from 8:00 to 9:30 pm, on the day before colonoscopy. The colonoscopy had to be performed the next morning, before 2:00 pm. Bowel preparation in the PEG-ASC–split assumption group consisted of 1 L of purgative solution followed by 0.5 L of clear liquids assumed twice, over a period of 90 min, respectively from 8:00 to 9:30 pm on the evening before colonoscopy, and from 7:00 to 8:30 am on the day of colonoscopy. Colonoscopy had to be performed in the afternoon. Bowel preparation in the SPMC arm - standard assumption, consisted of 150 ml of purgative solution followed by 1.5 L of clear liquids over a 90 min period, assumed twice, respectively at 2:00 pm and at 8:00 pm, on the day before colonoscopy. Colonoscopy had to be performed the next morning, before 2:00 pm. Bowel preparation in the SPMC–split assumption arm, consisted of 150 ml of purgative solution followed by 1.5 L of clear liquids over 90 min, assumed twice, respectively at 8:00 pm on the night before colonoscopy, and at 7:00 am on the day of colonoscopy. Colonoscopy had to be performed in the afternoon. In both arms, patients were asked to (i) follow a low-fiber diet and assume at least 2 L of water for 4 days before colonoscopy; (ii) assume a liquid diet on the day before colonoscopy and (iii) fast on the day of colonoscopy. All these instructions and the steps of bowel preparation were reviewed by the research nurse with each single subject at the time of enrollment.

The primary end-point of the study was the quality of bowel preparation. This was assessed according to the “Boston Bowel Preparation Scale” (BBPS), whose rating scale is between 0 and 3. The assessment was carried out prior to water flushing for the following colonic segments: right colon (caecum and ascending colon); transverse colon (including hepatic flexure and splenic flexure); left colon (descending and sigmoid colon, rectum). Consequently, the total score (0 to 3 for each segment) ranged from 0 to 9. Success was a total score between 6 and 9, with at least a score of 2 for each colonic segment (24, 25).

Colonoscopy was performed according to standard of practice of each participating center. All the exams were videotaped in their entirety with HD recorders and the CDs obtained were collected by the data manager of the Clinical Trial Unit for central blinded review. Five endoscopists (VDA, MDB, EDG, PM, and GBR) acted as blinded reviewers for the assessment of bowel preparation of the recorded colonoscopies in their totality. Each CD was reviewed and the quality of the bowel preparation was scored independently by two of them, who were selected by the trial coordinator at the Clinical Trial Unit. The chosen reviewers were unaware respectively of the bowel preparation assumed by the patient and the results of each other evaluation. Success was defined as a BBPS score ≥ 6, with at least a score of two for each colonic segment. In case of contrasting evaluation, a third reviewer was involved. In any case, a reviewer could not review an exam performed by her/himself. The outcome of each colonoscopy was described according to completeness of exploration (complete vs. incomplete) and duration time; causes of incomplete exploration were described.

Evaluation of patient acceptability of each bowel preparation was a co-primary end-point. The patients were provided a diary that was completed and returned to the research nurse before colonoscopy. The diary included visual analogic scales (VAS) from 0 to 10 (lower score representing a better outcome) and regarded the impact of purgative solutions on the following items: food intake (no impact up to inability to eat), taste (no impact up to very bad taste), simplicity of assumption (very simple up to very difficult), personal activity (no effect up to inability to perform personal activities), work activity (no effect up to inability to perform work activities), general perception (excellent up to very bad). Patients were also required to record whether all the purgative solution was assumed within the required time period and whether the correct amount of clear liquids was consumed. Finally, patients were asked if they were willing to repeat the same bowel preparation (no/yes). Compliance was calculated from patient diaries that inquired about the adherence to the prescribed diet, timing of assumptions, assumed volumes of both purgative solutions and clear liquids. Compliance was defined as full (all prescriptions respected), or less-than-full (one or more prescriptions not respected).

Statistical analysis was based on a modified intention-to-treat strategy (mITT), excluding patients who did not actually start bowel preparation. Success rate in the two arms was compared using the chi-square test. In the primary analysis, cases in which colonoscopy was interrupted for reasons other than bowel cleansing were considered failures. A sensitivity analysis was planned excluding those cases in which colonoscopy was interrupted before the evaluation of all three colonic segments for reasons independent of bowel cleansing (i.e., stricture of the colon). Success rate in the two arms was described for the subgroups defined according the meaningful clinical characteristics (gender, age, bowel segment, administration modality, and patients experience with a previous colonoscopy). No cutoff score for acceptability was defined in the protocol, outcomes of the scales in the two arms were compared as central measures by the Wilcoxon rank sum test. General perception was considered as a global outcome measure and the individual scores of the different dimensions were considered as secondary outcome measures. Compliance rate in the two arms was compared using the chi-square test.

## Results

**FIGURE 1:**
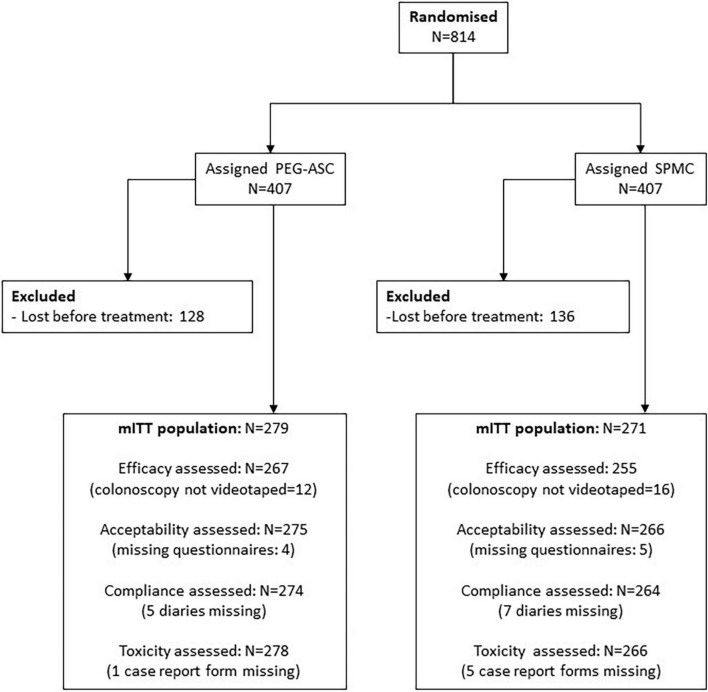
Patients flow.

From November 7th, 2011, to February 16th, 2015, 814 subjects were enrolled in seven Italian centers and assigned to PEG-ASC (*n* = 407) or SPMC (*n* = 407): they represented the ITT population. Overall, 264 subjects were excluded because they were lost before starting bowel preparation and the remaining 550 cases represented the modified ITT (mITT) population (Figure 1). Details of both ITT and mITT are summarized in Table 1. ITT and mITT populations were similar in terms of baseline characteristics (Table 1). In the mITT population, the median age was 54 (range 18—94); 297/550 (54%) individuals were males; 83/550 (15%) subjects had already undergone colonoscopy. The most frequent indication for colonoscopy was diagnosis (351/550, 64%), followed by screening (134/550, 24%) and follow-up after polypectomy (62/550, 12%). Two-thirds of the bowel preparations were performed according to standard modality in both study arms, while one third of the purgative solutions was administered with the split modality approach.

**TABLE 1:** Baseline data of intention-to-treat (ITT) and modified intention-to-treat (mITT) populations by assigned arm.

	Intention-to-treat		Modified intention-to-treat	
	PEG-ASC	SPMC	PEG-ASC	SPMC
	(*N* = 407)	(*N* = 407)	(*N* = 279)	(*N* = 271)
	n (%)	n (%)	n (%)	n (%)
**Gender, n (%)**				
Male	216 (53.1%)	208 (51.1%)	148 (53.0%)	149 (55.0%)
Female	191 (46.9%)	199 (48.9%)	131 (47.0%)	122 (45.0%)
**Age, years**				
Median	54	55	54	54
Range	18–86	18–94	18–86	18–94
IQR	45–66	44–66	44–66	44–64
Previous colonoscopy, n (%)	51 (12.5%)	60 (14.7%)	41 (14.7%)	42 (15.5%)
**Aim of the colonscopy, n (%)**				
Diagnosis	260 (63.9%)	259 (63.6%)	176 (63.1%)	175 (64.6%)
Screening	100 (24.6%)	102 (25.1%)	72 (25.8%)	62 (22.9%)
Follow-up after colonoscopy	47 (11.5%)	46 (11.3%)	31 (11.1%)	34 (12.5%)
**Scheme of administration, n (%)**				
Standard	252 (61.9%)	260 (63.9%)	181 (64.9%)	178 (65.7%)
Split	155 (38.1%)	147 (36.1%)	98 (35.1%)	93 (34.3%)

In the mITT population, 522 cases were assessible for efficacy evaluation because colonoscopy was not videotaped in 28 cases. There was no statistically significant difference in the rate of bowel cleansing according to BBPS between the two arms: in 252/267 (94.4%) subjects of PEG-ASC arm and 244/255 individuals (95.7%) of SPMC group overall colon cleansing was successful with a BBPS > 6, and a minimum score of 2 in each bowel segment (*P* = 0.49). Table 2 shows that the success rate of both bowel preparations was not influenced by gender, age, bowel segment, administration modality, and patients experience with a previous colonoscopy.

**TABLE 2:** Success of bowel preparation.

	PEG-ASC		SPMC		*P*
Overall success	252/267	94.4%	244/255	95.7%	0.49
**By gender**					
Males	134/141	95.0%	135/140	96.4%	
Females	118/126	93.7%	109/115	94.7%	
**By age**					
= 54	123/130	94.6%	118/123	95.9%	
> 54	129/137	94.2%	126/132	95.5%	
**By previous colonoscopy**					
No previous colonoscopy	217/229	94.8%	204/214	95.3%	
Already done previous colonoscopy	35/38	92.1%	40/41	97.6%	
**By administration scheme**					
Standard scheme	160/172	93.0%	157/165	95.2%	
Split scheme	92/95	96.8%	87/90	96.7%	
**By bowel side**					
Right bowel	252/267	94.4%	244/255	95.7%	
Transverse bowel	256/267	95.9%	249/255	97.6%	
Left bowel	266/267	99.6%	255/255	100%	

Success was defined as a Boston Bowel Prep Score > 6, with a minimum score of 2 in each of the 3 bowel segments.

Colonoscopy was reported less than complete in 40 cases, respectively 21/267 (8.0%) and 19/255 (7.7%) in the two arms (*P* = 0.86). These results were independent of the assumption modality of both bowel preparations. Among subjects assuming purgative solutions with standard modality, there was a success rate of 93.0% with PEG-ASC and 95.2% with SPMC, respectively; among subjects receiving the split dose, PEG-ASC was effective in 96.8% of cases and SPMC in 96.7% of patients, respectively. In the two arms, the duration of colonoscopy was comparable, with a median time of 21 min (interquartile range [IQR] 16—30) for procedure. Similarly, the withdrawal time of the scope during colonoscopy was equivalent with a median duration of 10 min in both study groups (IQR 8—15).

**FIGURE 2:**
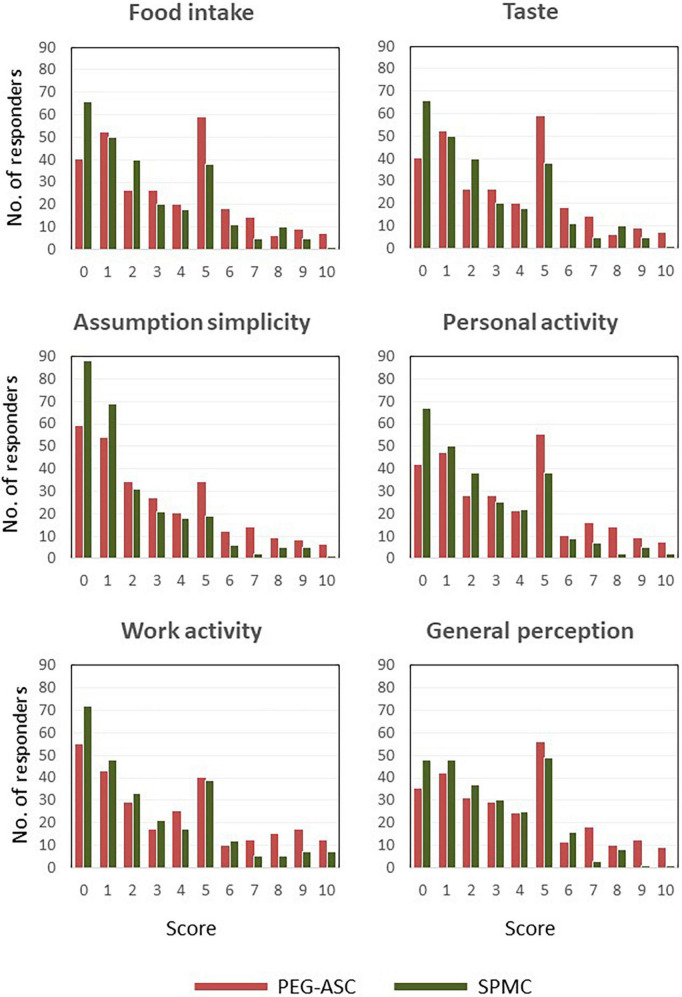
Score distribution.

The impact of bowel preparation on patient acceptance was significantly better with SPMC in all the explored scales. Perception of preparation had a mean score of 3.7 (SD ± 0.2) vs. 2.8 (SD ± 0.1) (*P* < 0.0001), respectively for PEG-ASC and SPMC. Details of mean (SD) scores for patient-reported effect on food intake, taste, ease of administration, working activities are summarized in Table 3. Graphical representations of score distributions are reported in Figure 2. The rate of patients willing to repeat the bowel preparation was significantly lower with PEG-ASC (81.3%), in comparison to SPMC (92.3%, *P* < 0.001).

**TABLE 3:** Impact of bowel preparation on patients’ acceptance (mean–SD).

	PEG - ASC *N* = 275		SPMC *N* = 266		*P*
Impact on food intake, mean (SD)	3.5	(0.2)	2.6	(0.1)	< 0.0001
Impact on taste, mean (SD)	3.6	(0.2)	2.3	(0.1)	< 0.0001
Easiness, mean (SD)	3.0	(0.2)	1.9	(0.1)	< 0.0001
Impact on daily activities, mean (SD)	3.5	(0.2)	2.5	(0.1)	< 0.0001
Impact on working activities, mean (SD)	3.6	(0.2)	2.7	(0.2)	< 0.0001
Perception of preparation, mean (SD)	3.7	(0.2)	2.8	(0.1)	< 0.0001

Compliance with assumption of purgative solutions and the subsequent amount of clear liquids was less than complete, respectively, in 18/274 (6.6%) subjects assuming PEG-ASC and in 26/264 (9.9%) individuals using SPMC bowel preparation (*P* = 0.17).

Side effects of bowel preparation by study arm are summarized in Table 4. Nausea and meteorism were significantly more bothersome with PEG-ASC than SPMC, while vomiting, abdominal cramps and anal irritation were comparable in both arms. Headache, chills, insomnia and asthenia were similar with both purgative solutions. Side effects were not influenced by the modality of assumption (i.e., standard vs. split) of both purgative solutions. There were no serious adverse events leading to discontinuation of bowel preparation in either group.

**TABLE 4:** Side effects of bowel preparation.

	PEG-ASC (*N* = 278)										SPMC (*N* = 266)										*P*
	Grade 0		Grade 1		Grade 2		Grade 3		Grade 4		Grade 0		Grade 1		Grade 2		Grade 3		Grade 4		
	*N*	(%)	*N*	(%)	*N*	(%)	*N*	(%)	*N*	(%)	*N*	(%)	*N*	(%)	*N*	(%)	*N*	(%)	*N*	(%)	
Nausea	242	(87.1%)	18	(6.5%)	13	(4.7%)	3	(1.1%)	2	(0.7%)	230	(86.5%)	30	(11.3%)	5	(1.9%)	1	(0.4%)	0	(0.0%)	0.048
Vomiting	265	(95.3%)	8	(2.9%)	2	(0.7%)	2	(0.7%)	1	(0.4%)	261	(98.1%)	3	(1.1%)	2	(0.8%)	0	(0.0%)	0	(0.0%)	0.28
Meteorism	237	(85.3%)	21	(7.6%)	12	(4.3%)	7	(2.5%)	1	(0.4%)	233	(87.6%)	26	(9.8%)	7	(2.6%)	0	(0.0%)	0	(0.0%)	0.047
Abdominal cramps	246	(88.5%)	22	(7.9%)	6	(2.2%)	3	(1.1%)	1	(0.4%)	242	(91.0%)	18	(6.8%)	6	(2.3%)	0	(0.0%)	0	(0.0%)	0.38
Anal discomfort	248	(89.2%)	16	(5.8%)	9	(3.2%)	4	(1.4%)	1	(0.4%)	242	(91.0%)	13	(4.9%)	7	(2.6%)	4	(1.5%)	0	(0.0%)	0.85
Headache	255	(91.7%)	17	(6.1%)	2	(0.7%)	3	(1.1%)	1	(0.4%)	243	(91.4%)	15	(5.6%)	7	(2.6%)	0	(0.0%)	1	(0.4%)	0.20
Asthenia	244	(87.8%)	30	(10.8%)	3	(1.1%)	0	(0.0%)	1	(0.4%)	233	(87.6%)	23	(8.6%)	8	(3.0%)	2	(0.8%)	0	(0.0%)	0.18
Chills	255	(91.7%)	14	(5.0%)	6	(2.2%)	2	(0.7%)	1	(0.4%)	251	(94.4%)	6	(2.3%)	7	(2.6%)	2	(0.8%)	0	(0.0%)	0.40
Insomnia	267	(96.0%)	8	(2.9%)	2	(0.7%)	0	(0.0%)	1	(0.4%)	256	(96.2%)	2	(0.8%)	7	(2.6%)	1	(0.4%)	0	(0.0%)	0.08

## Discussion

Accurate bowel cleansing and patient acceptability of bowel preparation are the two pillars of quality for colonoscopy (4, 7, 26–28). We chose these two parameters as co-primary endpoints of this trial that compares two low-volume purgative solutions, routinely used in clinical practice. The results of our study confirm that both SPMC and PEG-ASC are highly efficacious in terms of bowel cleansing, independently of the administration modality. Our findings are consistent with those of eight smaller randomized clinical trials which compared SPMC with PEG-ASC (Table 5; 11–18). However, these studies had a single-center design and evaluated a relatively small number of patients. The current study is a large prospective, randomized trial which was needed to add further and conclusive evidence that SPMC is as effective as PEG-ASC and shows better tolerability. Similarly, three recent meta-analyses showed that PEG-based purgative solutions and SPMC have comparable efficacy in bowel cleansing, either when assumed with standard modality or with split regimen (28–30). Moreover, a phase 3 clinical trial comparing a novel very low (1 L) PEG-based bowel preparation with SPMC showed that there is no difference in bowel cleansing efficacy between the two purgative solutions, when both are assumed with standard modality (31).

**TABLE 5:** Randomized trials comparing sodium picosulphate plus magnesium citrate (SPMC) with polyethylene glycol plus ascorbic acid (PEG-ASC).

Authors	Ref.	Country	Design	#pts (ITT/PP)	SPMC (ITT/PP)	PEG-ASC (ITT/PP)	Bowel cleansing	Patients compliance	Patients acceptance
Manes et al. (11)	11	Italy	Multicenter	293/285	145/140	148/145	75.7% SPMC Vs. 76.5% PEG-ASC	83.6% SPMC Vs. 77.9% PEG-ASC	*97.8% SPMC Vs. 83.4% PEG-ASC
Choi et al. (12)	12	South Korea	Single-center	220/200	110/102	110/98	88.2% SPMC Vs. 85.7% PEG-ASC	98% SPMC Vs. 99% PEG-ASC	83.3% SPMC Vs. 85.7% PEG-ASC
Jeon et al. (13)	13	South Korea	Single-center	388/356	193/165	195/191	90.3% SPMC Vs. 89.5% PEG-ASC	*87% SPMC Vs. 99% PEG-ASC	92.1% SPMC Vs. 90.6% PEG-ASC
Sahebally et al. (14)	14	Ireland	Single-center	130/130	64/64	66/66	75% SPMC Vs. 1.8% PEG-ASC	93.8% SPMC Vs. 92.4% PEG-ASC	*95.3% SPMC Vs. 84.9% PEG-ASC
Worthington et al. (15)	15	UK	Single-center	70/65	33/33	32/30	72.7% SPMC Vs. 84.4% PEG-ASC	100% SPMC Vs. 96.9% PEG-ASC	NR% SPMC Vs. NR% PEG-ASC
Yoo et al. (16)	16	South Korea	Single-center	200/200	100/100	100/100	80% SPMC Vs. 82% PEG-ASC	94% SPMC Vs. 88% PEG-ASC	NR% SPMC Vs. NR% PEG-ASC
Seo et al. (17)	17	South Korea	Single-center	223/223	114/114	109/109	93.8% SPMC Vs. 93.5% PEG-ASC	*84.2% SPMC Vs. 55.9% PEG-ASC	*92.1% SPMC Vs. 83.4% PEG-ASC
Mathus-Vliegen et al. (18)	18	Netherlands	Single-center	354/337	177/171	177/166	75.8% SPMC Vs. 81.4% PEG-ASC	NR% SPMC Vs. NR% PEG-ASC	94% SPMC Vs. 60% PEG-ASC
D’Angelo et al.		Italy	Multicenter	550/522	271/254	279/268	95.7% SPMC Vs. 94.4% PEG-ASC	90.1% SPMC Vs. 93.4% PEG-ASC	*92.8% SPMC Vs. 81.3% PEG-ASC

ITT = number of randomized patients (intention to treat); PP = number of treated patients (per protocol).

NR = % not reported in full text; *p < 0.05.

In both study arms, the quality of bowel preparation was not influenced by gender, age, bowel segment, administration modality, and previous colonoscopy (Table 2). However, an excellent bowel preparation (BBPS ≥ 8) was accomplished in only 13% of individuals, independently of the bowel preparation used. These results suggest that bowel cleansing is not influenced by the above-mentioned parameters, which are usually considered crucial for the effectiveness of bowel preparation in clinical trials. The majority of colonoscopies (82.5%) had satisfactory bowel cleansing (6 ≤ BBPS ≤ 7), which is consistent with the average evaluation of bowel cleansing usually documented in the majority of colonoscopies routinely performed in clinical practice.

The use of patient-reported questionnaires allowed us to assess patient perception of the impact of the two bowel preparations. Indeed, SPMC was significantly better than PEG-ASC in all the domains explored: food intake, simplicity of assumption, personal activity, work activity, and general perception. In addition, the number of patients willing to repeat bowel preparation was significantly higher with SPMC than with PEG-ASC, even if patient completion rate was similar for both purgative solutions. Although the real volume of solution ingested was not recorded in the patient diaries, it was requested to report complete vs. incomplete assumption of purgative solutions. This information correlated with the success of bowel preparation, which was obtained in 95% of subjects assuming the entirety of either one or the other purgative solution, independently of assumption modality (i.e., standard vs. split). We acknowledge that there are data showing that compliance may not be linked with the volume of purgative solutions but the routine use of low volume purgative solutions for bowel preparation in clinical practice supports our results (32). Volume reduction and improved palatability of purgative solutions increase patient compliance for bowel preparation before colonoscopy, despite the fact that they still have to assume an additional volume (2–3 L) of clear liquids, as recommended to the patients enrolled in the current study (1, 2). This was evident for both PEG-ASC and SPMC, with the latter being more acceptable and preferred by the patients recruited in the trial. Similar results were reported by the majority of the studies which compared SPMC to PEG-ASC: SPMC was usually rated a favorable purgative solution, with good taste and easability for consumption (11, 13–20). Only one study reported that patient overall tolerance was higher for PEG-ASC than SPMC (12). These differences might be due to several reasons, including patients age, race or prior use of specific bowel cleansing solutions.

In the current study, the type and the incidence of adverse events were similar to those reported in other studies and they were independent of the modality of assumption. The most frequent adverse events were gastrointestinal symptoms, with nausea and meteorism being significantly bothersome in patients assuming PEG-ASC. No patients reported adverse events resulting from dehydration, which can result in electrolyte imbalance and hypotension in elderly and/or frail subjects assuming SPMC (13, 16, 17, 19, 20). According to a recent meta-analysis, SPMC is not recommended in patients with renal insufficiency, end-stage liver disease, heart failure and electrolyte abnormalities (30). These relative contraindications could limit the use of SPMC for bowel preparation in an open access system, even if a recent study reported no SPMC related adverse events in a physically disabled population (18).

Our study has some strengths that are worth emphasizing. First, this is one of the largest trial comparing PEG-ASC with SPMC, and its results are comparable in terms of efficacy and safety with those of other smaller randomized trials conducted in both Eastern and Western countries (11–18). Second, the evaluation of the primary endpoint was done by two independent blind reviewers who reviewed each single recorded colonoscopy in its entirety. Therefore, the bowel preparation was scored without the possible bias of the endoscopist who performed the colonoscopy, ensuring the quality of the results and the reliability of our findings. To our knowledge, a similarly robust study design was planned only in another randomized trial (22). Third, PEG-ASC is the most used low-volume PEG purgative solution and it has clearly shown to have similar efficacy and better tolerability than high-volume PEG-ELS solution (5, 6, 26). Therefore, the comparison of SPMC with PEG-ASC is a warranty of non-inferiority for SPMC, which is equally effective as bowel preparation. Forth, the evaluation of patient’s acceptance of bowel preparation has been thoroughly investigated in the current study, allowing to conclude that SPMC is better tollerated and accepted than low–volume PEG solutions with few exceptions (12). Volume reduction and palatability of SPMC increases patient compliance for this bowel preparation, despite the fact that they still have to assume an additional volume (2–3 L) of clear liquids (1, 2). Finally, the study was conducted in a manner that reflects current clinical practice, since colonoscopies were performed according to the standard practice of each participating center which used different modalities of execution of the exam (e.g., different regimen of sedation, single or double operator, different brands of endoscopes).

On the other hand, our study has several limitations that merit discussion. First, this study was affected by a larger (30%) than expected dropout of enrolled subjects due to the fact that at the time of the study patients were allowed to book the colonoscopy simultaneously at different institutions and subsequently decide where to undergo the procedure, on the basis of the fastest waiting list. This caused a longer than planned duration of enrollment and forced us to apply a modified-intention-to-treat strategy for the analysis. Second, we are publishing our data quite late after the end of the enrollment: this was substantially due to the time necessary for collecting the blinded revisions required by the study design, and the subsequent delay in the evaluation of the data. Third, we did not randomize the modality of assumption of bowel preparation; the majority of our patients received the bowel preparation in standard modality and this is not consistent with the routine use of split regimen. The latter has been shown to be superior, regardless of type of bowel preparation and dose (33). Our choice aimed to simplify patient management at participating centers, since there is no hypothesis of interaction between the modality of assumption and the type of bowel preparation (28–30). We recognize that our findings of comparable results in terms of bowel cleanness with standard and split preparations are limited because of lack of randomization and tend to be inconsistent with the literature data that favors split modality (2). However, we believe that the reliability of our findings are warranted by the blinded revision on which our results are based. Fourth, at the time of the execution of the study, it was standard of practice to recommend a low-fiber diet for 4 days before colonoscopy and the assumption of a liquid diet on the day before colonoscopy. Nowadays, patients are instructed to follow a regular diet until lunch on the day before colonoscopy (2). However, the results of our study were not affected by the quality of the diet assumed by the patients in the days preceding the colonscopy. Fifth, the effects of the two regimens on intravascular volume and electrolyte balance were not addressed in the current study, but previous studies have demonstrated that SPMC is safe if high risk patients are excluded (16, 19, 20). Finally, adenoma detection rate was not recorded. However, the definition of success according to BBPS is the most important premise for optimal colonoscopy and we decided to use only BBPS for the comparison of the two bowel preparations (24, 25).

In conclusion, the most significant finding of this study is the validation of previous results by means of a large, prospective, randomized, multicenter trial which confirms that SPMC is equally effective and shows increased tolerability in comparison with PEG-ASC. Therefore, SPMC could be used as an alternative to standard low-volume PEG based purgative solutions for bowel preparation in adult, healthy outpatients who do not tolerate either high or low-volume purgative solutions.

## Data availability statement

The datasets presented in this study can be found in online repositories. The names of the repository/repositories and accession number(s) can be found below: 10.5281/zenodo.6957443.

## Ethics statement

The studies involving human participants were reviewed and approved by Comitato Etico dell ‘IRCCS Istituto Nazionale per lo Studio e la Cura dei Tumori Fondazione Giovanni Pascale di Napoli. The patients/participants provided their written informed consent to participate in this study.

## Author contributions

VDA, MDM, MCP, and MDB managed the overall project. FP and CG analyzed all the data. MDB and FP prepared the first draft and finalized the article on the basis of comments from the other authors. CG, MDM, MCP, and VDA reviewed the first draft. All other authors provided data, reviewed results and contributed to the final version of the article.

## Footnotes

1
http://usc-intnapoli.net
