# Supplementary material for: A multicenter randomized phase 4 trial comparing sodium picosulphate plus magnesium citrate vs. polyethylene glycol plus ascorbic acid for bowel preparation before colonoscopy. The PRECOL trial
Source: Front Med (Lausanne). 2022 Dec 8;9:1013804. doi: 10.3389/fmed.2022.1013804 (PMC9773881; doi:10.3389/fmed.2022.1013804)
Supplement: Supplementary file 1 [file Data_Sheet_1.PDF]

SODIO PICOSOLFATO E MAGNESIO CITRATO VERSO POLYETHYLENE GLYCOLE (PEG) NELLA PREPARAZIONE INTESTINALE PER LA COLONSCOPIA: STUDIO RANDOMIZZATO DI FASE IV

Promotore dello studio:
Istituto Nazionale per lo Studio e la Cura dei Tumori di Napoli

Ricercatori principali:
dr. Valentina D'Angelo
dr. Mario de Bellis
Unità Operativa Complessa Endoscopia Diagnostica ed Operativa
Istituto Nazionale per lo Studio e la Cura dei Tumori di Napoli

Ricercatori associati:
dr. Elena Di Girolamo
dr. Pietro Marone
dr. Giovanni Battista Rossi
dr. Alfonso Tempesta
Unità Operativa Complessa Endoscopia Diagnostica ed Operativa
Istituto Nazionale per lo Studio e la Cura dei Tumori di Napoli
Centri SIED partecipanti

Coordinamento dello studio:
Unità Sperimentazioni Cliniche
Istituto Nazionale per lo Studio e la Cura dei Tumori di Napoli
dr. Massimo Di Maio
dr.ssa Maria Carmela Piccirillo
dr. Francesco Perrone

Analisi statistica
Cattedra di Statistica Medica
Seconda Università di Napoli
dr.ssa Simona Signoriello
prof. Ciro Gallo

INDICE

INDICE 2
SINOSSI..3
Premesse e razionale dello studio..5
ObiettivI dello studio 6
Obiettivo primario dello studio 6
Obiettivi secondari dello studio...6
Disegno dello studio 6
SELEZIONE DEI PAZIENTI 7
Criteri di inclusione 7
Criteri di esclusione 7
Modalità, tempi e criteri di somministrazione DELLA PREPARAZIONE INTESTINALE 8
Braccio 1 8
INDIRIZZI UTILI...18
BIBLIOGRAFIA..19

SINOSSI

Obiettivo primario dello studio

- Confrontare **l'efficacia**, in termini di qualità della preparazione intestinale, del lassativo a base di sodio picosolfato e magnesio citrato (NapP) rispetto alla classica preparazione con PEG, in pazienti sottoposti a pancolonscopia diagnostica.
- Confrontare **il grado di accettabilità** della preparazione intestinale con NapP rispetto alla preparazione con PEG.

Obiettivi secondari dello studio

- Confrontare **la compliance** alla preparazione intestinale con NapP rispetto alla preparazione con PEG.

DISEGNO DELLO STUDIO

Studio multicentrico di fase IV, prospettico, randomizzato, a due bracci (1:1).

Lo studio verrà condotto **in aperto** per quanto riguarda l'assegnazione del trattamento, in quanto sia l'esecutore della pancolonscopia che i soggetti ad essa sottoposti saranno a conoscenza del tipo di preparazione assegnata dalla randomizzazione.

La valutazione della qualità della preparazione intestinale verrà condotta **in cieco**, in quanto tale valutazione avverrà mediante analisi della registrazione dell'esame da parte di due sperimentatori, diversi da quello che ha eseguito l'esame, che non saranno a conoscenza del tipo di preparazione assegnata a quel soggetto.

SELEZIONE DEI PAZIENTI

Criteri di inclusione

- Soggetti candidati a effettuare pancolonscopia:
  - a scopo diagnostico,
  - di screening,
  - di follow up post polipectomia
- Consenso informato
- Età superiore a 18 anni

Criteri di esclusione

- Soggetti precedentemente sottoposti a resezione colica
- Diagnosi di malattia infiammatoria intestinale (*IBD*) severa
- Diagnosi di insufficienza renale

- Diagnosi di insufficienza cardiaca (classi NYHA III e IV)
- Diagnosi di insufficienza epatica (classe B o C secondo la classificazione di Child-Pugh)
- Stato di severa disidratazione
- Assunzione di litio
- Gravidanza
- Presenza di controindicazioni note alla preparazione intestinale (es. ipermagnesemia, rabdomiolisi)
- Presenza di controindicazioni note all’esecuzione della pancolonscopia

DIMENSIONAMENTO DEL CAMPIONE

Lo studio è dimensionato con l’obiettivo di riconoscere una differenza del 10% (considerata come valore minimo clinicamente rilevante) nella proporzione di successi (adeguata preparazione intestinale) tra le due modalità di preparazione confrontate (*endpoint primario*).

Considerando un tasso di successo nel braccio inferiore pari all’80%, un errore alfa biilaterale pari a 0.05, lo studio garantirà il 90% di potenza nell’evidenziare la differenza prevista del 10%, con l’arruolamento di 525 pazienti.

PREMESSE E RAZIONALE DELLO STUDIO

- Per una corretta preparazione del paziente alla colonscopia è indispensabile una buona pulizia intestinale tale da permettere una completa visualizzazione della mucosa colica (90-100%). Affinché la preparazione intestinale sia ottimale, è prevista l'assunzione di soluzioni saline in grandi quantità (4 litri), in un tempo relativamente breve. Questo tipo di procedura non risulta accettata di buon grado dai pazienti, che hanno difficoltà o addirittura impossibilità a completare la preparazione, riportando inevitabili conseguenze negative sulla qualità della pulizia dell'intestino e di conseguenza sulla stessa resa diagnostica della colonscopia.

- Nel tentativo di migliorare la compliance dei pazienti per la preparazione intestinale, è stato ridotto il volume di soluzione salina (viene solitamente utilizzato il polyethylene glycole - PEG) da 4 a 2 litri. Tale approccio ha prodotto risultati soddisfacenti sia per la compliance del paziente, sia per il livello di pulizia del colon con una buona resa diagnostica della colonscopia. Tuttavia, un numero relativamente elevato di pazienti (20%), non riesce ad assumere la soluzione, anche a volumi ridotti, e a completare la preparazione intestinale necessaria per una colonscopia di qualità.

- Il sodio monofosfato, assunto in tre somministrazioni, diluito in circa 500 cc di acqua, consente di ottenere una discreta preparazione intestinale in quei pazienti che non tollerano elevati volumi di soluzione. Tuttavia, questo lassativo può avere seri effetti collaterali nei pazienti con patologie cardiache e renali, tanto da sconsigliarne l'uso, in questi casi, per la preparazione nei pazienti da sottoporre alla colonscopia.

- Recentemente è stato introdotto in Italia un lassativo a base di sodio picosolfato e magnesio citrato (NapP), utilizzato per la preparazione intestinale nel Regno Unito. Il sodio picosolfato è un purgante stimolante ed il magnesio citrato è un lassativo osmotico, con una azione aggiuntiva di stimolazione del rilascio di colecistochinina. I due principi attivi agiscono sinergicamente trattenendo acqua nel lume intestinale e stimolando la peristalsi. Questa associazione ha il vantaggio di ridurre il volume della soluzione dal gusto salino (all'incirca 300 cc), con il vantaggio di una migliore tollerabilità da parte del paziente.

OBIETTIVI DELLO STUDIO

Obiettivo primario dello studio

- Confrontare **l'efficacia**, in termini di qualità della preparazione intestinale, del lassativo a base di sodio picosolfato e magnesio citrato (NapP) rispetto alla classica preparazione con PEG, in pazienti sottoposti a pancolonscopia diagnostica.
- Confrontare **il grado di accettabilità** della preparazione intestinale con NapP rispetto alla preparazione con PEG.

Obiettivi secondari dello studio

- Confrontare **la compliance** alla preparazione intestinale con NapP rispetto alla preparazione con PEG.

DISEGNO DELLO STUDIO

Studio multicentrico di fase IV, prospettico, randomizzato, a due bracci (1:1).

Lo studio verrà condotto **in aperto** per quanto riguarda l'assegnazione del trattamento, in quanto sia l'esecutore della pancolonscopia che i soggetti ad essa sottoposti saranno a conoscenza del tipo di preparazione assegnata dalla randomizzazione.

La valutazione della qualità della preparazione intestinale verrà condotta **in cieco**, in quanto tale valutazione avverrà mediante analisi della registrazione dell'esame da parte di due sperimentatori, diversi da quello che ha eseguito l'esame, che non saranno a conoscenza del tipo di preparazione assegnata a quel soggetto.

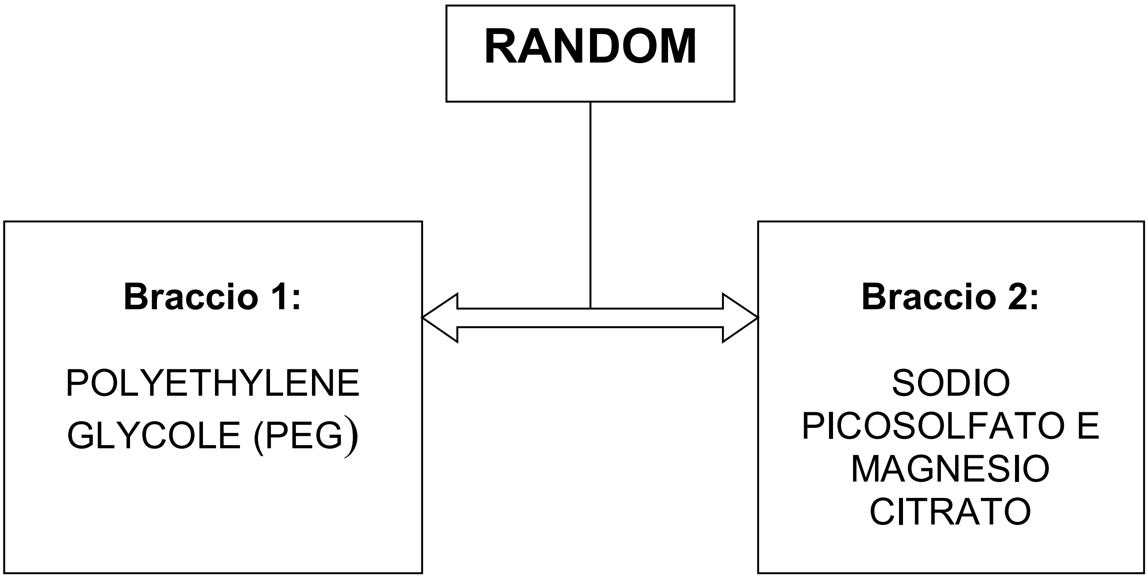

SELEZIONE DEI PAZIENTI

Criteri di inclusione

- Soggetti candidati a effettuare pan colonscopia:
    - a scopo diagnostico,
    - di screening,
    - di follow up post polipectomia
- Consenso informato
- Età superiore a 18 anni

Criteri di esclusione

- Soggetti precedentemente sottoposti a resezione colica
- Diagnosi di malattia infiammatoria intestinale (*IBD*) severa
- Diagnosi di insufficienza renale
- Diagnosi di insufficienza cardiaca (classi NYHA III e IV)
- Diagnosi di insufficienza epatica (classe B o C secondo la classificazione di Child-Pugh)
- Stato di severa disidratazione
- Assunzione di litio
- Gravidanza
- Presenza di controindicazioni note alla preparazione intestinale (es. ipermagnesemia, rabdomiolisi)
- Presenza di controindicazioni note all’esecuzione della pancolonscopia

MODALITÀ, TEMPI E CRITERI DI SOMMINISTRAZIONE DELLA PREPARAZIONE INTESTINALE

Entrambi i trattamenti per la preparazione intestinale confrontati in questo protocollo sono disponibili per l'uso nella pratica clinica.

La somministrazione della preparazione intestinale potrà avvenire, sia nel braccio 1 (PEG) che nel braccio 2 (NapP) con modalità standard (preparazione eseguita interamente la sera prima dell'esame) oppure con modalità split (preparazione eseguita per metà la sera prima dell'esame e per metà la mattina dell'esame), a discrezione dello sperimentatore. La modalità di assunzione (standard o split), dichiarata al momento della randomizzazione, sarà tra le variabili di minimizzazione della procedura (vedi paragrafo Randomizzazione).

Braccio 1 (PEG):

Assunzione standard

- ore 17.00-18.30 del pomeriggio precedente la colonscopia
  - il soggetto assumerà 1 litro di soluzione seguito da ½ litro di liquidi a piacere del paziente
- ore 20.00-21.30 della sera precedente la colonscopia
  - il soggetto assumerà 1 litro di soluzione seguito da ½ litro di liquidi a piacere del paziente
- La colonscopia potrà essere eseguita nel mattino successivo, entro le 14.00

Assunzione split

- ore 20.00-21.30 del pomeriggio precedente la colonscopia
  - il soggetto assumerà 1 litro di soluzione seguito da ½ litro di liquidi a piacere del paziente
- ore 07.00-08.30 del mattino della colonscopia
  - il soggetto assumerà 1 litro di soluzione seguito da ½ litro di liquidi a piacere del paziente
- La colonscopia potrà essere eseguita nel pomeriggio successivo, entro le 20.00

Dieta:

- Nei quattro giorni precedenti l'esecuzione della preparazione intestinale:
  - dieta povera di fibre;
  - bere almeno 2 litri di acqua al dì
- Il giorno della preparazione intestinale:
  - Dieta liquida
- Giorno dell'esecuzione della colonscopia:
  - digiuno

Braccio 2 (NapP):

Assunzione standard

- ore 14.00 del pomeriggio precedente la colonscopia:
    - il soggetto assumerà 150 ml. di soluzione;
- dalle ore 14.00 alle ore 15:00 – 15:30 del pomeriggio precedente la colonscopia:
    - il soggetto assumerà 1 - 1,5 litri di liquidi a piacere;
- ore 20.00 del pomeriggio precedente la colonscopia:
    - il soggetto assumerà 150 ml. di soluzione;
- dalle ore 20.00 alle ore 21:00 – 21:30 del pomeriggio precedente la colonscopia:
    - il soggetto assumerà 1 - 1,5 litri di liquidi a piacere,
- La colonscopia potrà essere eseguita nel mattino successivo, entro le 14.00

Assunzione split

- ore 20.00 del pomeriggio precedente la colonscopia:
    - il soggetto assumerà 150 ml. di soluzione;
- dalle ore 20.00 alle ore 21-00 - 21:30 del pomeriggio precedente la colonscopia:
    - il soggetto assumerà 1 - 1,5 litri di liquidi a piacere;
- ore 7.00 del mattino della colonscopia:
    - il soggetto assumerà 150 ml. di soluzione;
- dalle ore 7.00 alle ore 8:00 - 8:30 del mattino della colonscopia:
    - il soggetto assumerà 1 - 1,5 litri di liquidi a piacere;
- La colonscopia potrà essere eseguita nel pomeriggio successivo, entro le 20.00

Dieta:

- Quattro giorni precedenti:
    - dieta povera di fibre;
    - bere almeno 2 litri di acqua al dì
- Il giorno della preparazione intestinale:
    - Dieta liquida
- Giorno dell'esecuzione dell'esame:
    - digiuno

VALUTAZIONE DELLA QUALITÀ DELLA PREPARAZIONE INTESTINALE

La valutazione della qualità della preparazione intestinale rappresenta **l'endpoint primario** dello studio.

La valutazione avverrà in cieco, in quanto eseguita da due sperimentatori che prenderanno comune visione della registrazione di ciascun esame, non essendo a conoscenza del tipo di preparazione assegnato al soggetto.

La qualità della preparazione intestinale sarà valutata in accordo alla "Boston Bowel Preparation Scale" (BBPS). Tale scala prevede un punteggio compreso tra 0 e 3:

- 0: mucosa non visibile a causa della presenza di feci solide che non possono essere rimosse (vedi pannello A della figura)
- 1: mucosa visibile solo in parte a causa della presenza di feci solide e/o liquide (vedi pannello B della figura)
- 2: mucosa visibile nonostante la presenza di minimi residui fecali, aspirabili. (vedi pannello C della figura)
- 3: mucosa ben visibile, senza residui fecali (vedi pannello D della figura).

The Boston bowel preparation scale — Lai et al

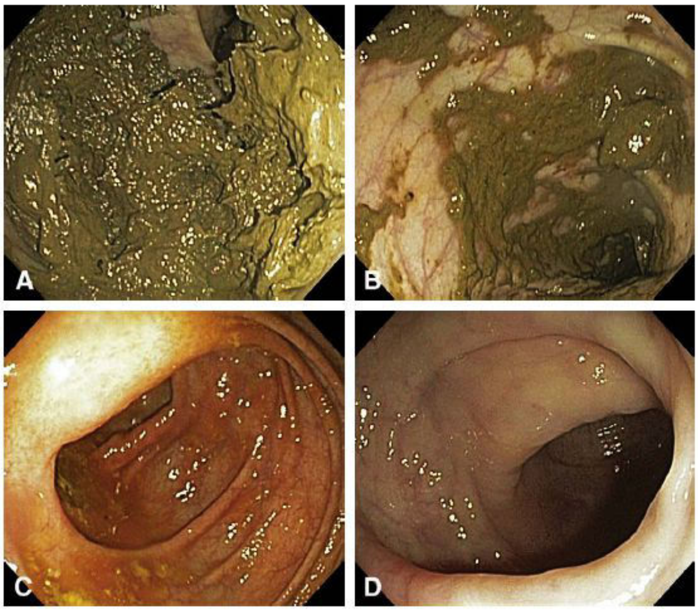

Figure 1. The BBPS. **A,** Segment score 0, unprepared colon segment with mucosa not seen because of solid stool that cannot be cleared. **B,** Segment score 1, portion of mucosa of the colon segment seen, but other areas of the colon segment not well seen because of staining, residual stool, and/or opaque liquid. **C,** Segment score 2, minor amount of residual staining, small fragments of stool and/or opaque liquid, but mucosa of colon segment seen well. **D,** Segment score 3, entire mucosa of colon segment seen well with no residual staining, small fragments of stool and/or opaque liquid.

- La valutazione della qualità secondo la suddetta scala sarà effettuata per i seguenti segmenti:
    - Colon destro (cieco e colon ascendente);
    - Colon trasverso (comprese la flessura epatica e la flessura splenica)
    - Colon sinistro (colon discendente, sigma, retto)
- Il punteggio complessivo potrà quindi essere compreso tra 0 e 9.
- Viene definito **successo** della preparazione intestinale **un punteggio complessivo compreso tra 6 e 9, con un punteggio non inferiore a 2 in ogni singolo segmento**.
- Esempi:
    - Colon destro 2, colon trasverso 2, colon sinistro 2: punteggio complessivo 6, successo (punteggio soddisfacente in tutti i segmenti).
    - Colon destro 1, colon trasverso 3, colon sinistro 2: punteggio complessivo 6, insuccesso (punteggio non soddisfacente per il colon destro)
    - Colon destro 1, colon trasverso 3, colon sinistro 3: punteggio complessivo 7, insuccesso (punteggio non soddisfacente per il colon destro)

VALUTAZIONE DELL'ACCETTABILITÀ DELLA PREPARAZIONE INTESTINALE

- Al momento dell'inserimento del soggetto in studio, dopo aver fornito al soggetto le informazioni relative allo studio ed aver ottenuto il consenso informato, si fornirà al paziente, insieme alla preparazione e alle relative istruzioni, un diario da compilare.
- Il giorno dell'esame, il paziente fornirà il consenso informato all'esecuzione della pancolonscopia e, prima di essere sottoposto all'esame, consegnerà all'infermiere e/o al medico il diario compilato durante la preparazione intestinale con l'obiettivo di valutare la sua compliance verso la soluzione utilizzata.
- Il diario compilato dal paziente al termine della procedura riguarderà le seguenti informazioni:
  - percentuale della dose assunta (100%, 75% o meno)
  - rispetto dei tempi di assunzione
  - dieta (VAS da 0 – eccellente - a 10 - pessimo)
  - gusto (VAS da 0 – eccellente - a 10 - pessimo)
  - semplicità (VAS da 0 – eccellente - a 10 - pessimo)
  - effetti sulla attività personale (VAS da 0 – nessuno - a 10 – attività impossibile)
  - effetti sulla attività lavorativa (VAS da 0 – nessuno - a 10 – attività impossibile)
  - disponibilità alla eventuale necessità di ripetizione dell'indagine con la stessa preparazione
  - percezione generale (VAS da 0 – eccellente - a 10 – pessima)
  - precedenti colonscopie (sì / no; data dell'ultima colonscopia)

VALUTAZIONE DEGLI EVENTI AVVERSI

Definizioni

Viene definito **evento avverso** qualsiasi evento indesiderato (sintomo, segno, nuova patologia, riscontro di una alterazione di laboratorio, ecc…) che si realizzi in un paziente inserito nello studio, indipendentemente dalla sua relazione con i farmaci in studio e dal braccio di trattamento assegnato.

Viene definito **evento avverso grave** (e quindi soggetto alla segnalazione rapida secondo le norme descritte più avanti) un evento avverso che consiste o esita in morte, pericolo di vita, ospedalizzazione oppure prolungamento del ricovero in ospedale, un'invalidità o un'incapacità grave o prolungata, un'anomalia congenita o un difetto alla nascita, necessità di intervento medico d'urgenza.

Raccolta e trasmissione degli eventi avversi gravi

Tutti gli eventi avversi gravi che si verifichino nel corso dello studio e nei 30 giorni successivi all'interruzione del trattamento dovranno essere raccolti e trasmessi utilizzando la **scheda di segnalazione eventi avversi gravi** (SEAG – vedi allegati).

La SEAG dovrà essere inviata per fax alla Segreteria dello studio entro il termine massimo di 2 giorni lavorativi dal momento in cui lo Sperimentatore è venuto a conoscenza dell'evento.

Indipendentemente dalla eventuale segnalazione mediante SEAG, tutti gli eventi avversi considerati correlati all'uso dei farmaci in studio e che configurino una forma di tossicità dovranno essere riportati nelle schede di raccolta dei dati sulla tossicità dei trattamenti.

CODIFICA DEL NESSO CAUSALE TRA TRATTAMENTO ED EVENTO

INESISTENTE: l'evento avverso non è collegato all'uso del farmaco

IMPROBABILE: l'evento avverso è dovuto ad una causa alternativa più probabile (i.e. farmaci o malattie concomitanti), e/o la relazione temporale suggerisce come improbabile la relazione causale.

POSSIBILE: l'evento avverso potrebbe dipendere dall'uso del farmaco, non essendoci evidenze conclusive di cause alternative ed essendo plausibile la relazione temporale, una relazione causale non può essere esclusa.

PROBABILE: l'evento avverso potrebbe dipendere dall'uso del farmaco, essendo supportato dalla relazione temporale e non essendo verosimili altre cause.

CERTO: l'evento avverso è conosciuto come possibile reazione avversa del farmaco in studio e non ci sono ragionevoli spiegazioni alternative.

DIMENSIONAMENTO DEL CAMPIONE

Lo studio è dimensionato con l'obiettivo di riconoscere una differenza del 10% (considerata come valore minimo clinicamente rilevante) nella proporzione di successi (adeguata preparazione intestinale) tra le due modalità di preparazione confrontate (*endpoint primario*).

Considerando un tasso di successo nel braccio inferiore pari all'80%, un errore alfa biilaterale pari a 0.05, lo studio garantirà il 90% di potenza nell'evidenziare la differenza prevista del 10%, con l'arruolamento di 525 pazienti.

ANALISI STATISTICA

La compliance verrà accuratamente descritta e confrontata tra i due bracci.

L'analisi di confronto di efficacia verrà condotta sulla base dell'*intention-to-treat.* Il confronto statistico verrà effettuato con il test del chi-quadro. Nell'analisi primaria, i casi in cui la colonscopia venga interrotta per motivi diversi dalla pulizia intestinale verranno considerati insuccessi.

Una analisi di *sensitivity* verrà condotta escludendo i casi in cui l'esame colonscopico verrà interrotto prima della valutazione dei 3 segmenti previsti per motivi indipendenti dalla pulizia intestinale (es. lesioni stenosanti).

PROCEDURE DI REGISTRAZIONE, RANDOMIZZAZIONE E RACCOLTA DATI

- Le procedure di registrazione, randomizzazione e raccolta dati saranno realizzate presso l'Unità Sperimentazioni Cliniche dell'Istituto Nazionale Tumori di Napoli.
- La registrazione è attraverso il sito web (http://usc-intnapoli.net).
- La randomizzazione verrà gestita centralmente dalla segreteria dello studio mediante una procedura automatica (disponibile al sito web http:\\usc-intnapoli.net) di minimizzazione che includerà come variabili di stratificazione:
    - il centro;
    - il motivo per cui è indicata la colonscopia (screening vs. precedente sospetto clinico o radiologico di neoplasia vs. altro);
    - l'aver praticato o meno precedentemente esami colonscopici;
    - la modalità di somministrazione della preparazione intestinale (assunzione standard vs. assunzione *split*).
- Lo stesso paziente non può essere arruolato due volte nello studio (nel caso di ripetizione di esame colonscopico).
- La raccolta dati sarà via web.

Recapiti per la registrazione e la randomizzazione:

Unità Sperimentazioni Cliniche

Istituto Nazionale per lo Studio e la Cura dei Tumori di Napoli

http://www.usc-intnapoli.net

e-mail: datamanager@usc-intnapoli.net

telefono: 081-5903571 fax: 081-7702938

tutti i giorni lavorativi (escluso il sabato) ore 9.30 – 15.30

INFORMAZIONE DEI SOGGETTI PARTECIPANTI E CONSENSO

- Prima della registrazione, tutti i soggetti potenzialmente eleggibili dovranno ricevere le informazioni complete sullo studio.
- Per poter essere registrati sarà necessario che i pazienti diano il consenso alla trattazione dei dati personali in forma anonima ed aggregata, ai sensi della legge 196/2003, e che sottoscrivano il modulo di consenso per la partecipazione allo studio.

PROCEDURE PER LA TUTELA DELLA *PRIVACY*

Allo scopo di tutelare la *privacy* dei pazienti inseriti nello studio si stabilisce quanto segue:

- Il Centro di coordinamento, che ha la responsabilità centralizzata delle procedure di registrazione, randomizzazione, raccolta e gestione dei dati, non fornirà ad altri i nominativi dei pazienti inseriti nello studio, tranne che agli Enti pubblici previsti dalla normativa vigente per finalità ispettive e di controllo (Ministero della Sanità, Comitati Etici);
- Successivamente alla registrazione verrà assegnato ai pazienti un codice numerico identificativo univoco (dal momento che ad ogni paziente inserito nello studio corrisponderà un numero progressivo) che verrà riportato insieme al codice identificativo del centro nella intestazione di tutte le schede raccolta dati (che potranno quindi essere anche inviate per fax) e che verrà utilizzato per tutte le comunicazioni riguardanti il singolo paziente;
- Una lista di decodifica del codice paziente esisterà esclusivamente presso i singoli centri partecipanti e presso la Segreteria dello studio.

ASPETTI AMMINISTRATIVI

- Lo studio è spontaneo, non sponsorizzato dalle aziende farmaceutiche produttrici dei presidi in sperimentazione.
- I presidi sono a carico del SSN perché in indicazione.
- Poichè le dosi previste sono uguali a quelle utilizzate come preparazione standard non vi è aggravio di spesa per il SSN.
- Una convenzione verrà stipulata tra il centro coordinatore ed ogni centro partecipante.
- Entrambe le preparazioni oggetto dello studio sono attualmente presenti in commercio ed utilizzate nella pratica clinica. Pertanto, non è stata stipulata specifica polizza assicurativa.
- La sperimentazione viene condotta in accordo con quanto previsto dalla normativa corrente, ai sensi dell'art.6 del Decreto Ministeriale del 17 dicembre 2004 (sperimentazione finalizzata al miglioramento della pratica clinica).

INDIRIZZI UTILI

Ricercatori principali:

Dr. Valentina D'Angelo, Dr. Mario de Bellis

UOC Endoscopia Diagnostica ed Operativa

Istituto Nazionale per lo Studio e la Cura dei Tumori di Napoli

Tel: 081 5903228

Fax: 081 5903804

Email: statrosa@alice.it
madebellis@alice.it

Centro coordinatore dello studio

Unità Sperimentazioni Cliniche

Istituto Nazionale per lo Studio e la Cura dei Tumori di Napoli

Dr. Massimo Di Maio, Dr. Francesco Perrone

Tel: 081 - 5903571

Fax: 081 - 7702938

Email: massimo.dimaio@usc-intnapoli.net
francesco.perrone@usc-intnapoli.net

Responsabile statistico:

Prof. Ciro Gallo

Cattedra Statistica Medica

Seconda Università di Napoli

E-mail: ciro.gallo@unina2.it

BIBLIOGRAFIA

1. RM NESS et al Predictors of inadeguate preparation for colonoscopy. Am J Gastroenterol 2001;96:1797-802

2. DH REX et al ASGE/ACG taskforce on quality in endoscopy. Quality indicators for colonoscopy. Gastrointest Endoscopy 2006;63(4):S16

3. A BITOUN et al Results of a prospective randomised multicentre controlled trial comparing a new 2-L ascorbic acid plus polyethylene glycol and electrolyte solution vs. sodium phosphate solution in patients undergoing elective colonoscopy. Aliment Pharmacol Ther 2006 ; 24: 1631–1642

4. C ELL et al Randomized Trial of Low-Volume PEG Solution *Versus* Standard PEG + Electrolytes for Bowel Cleansing Before Colonoscopy. Am J Gastroenterol 2007;102:1–11

5. J WORTHINGTON et al A randomised controlled trial of a new 2 litre polyethylene glycol solution versus sodium picosulphate + magnesium citrate solution for bowel cleansing prior to colonoscopy. Current Medical Research and Opinion 2008; Vol 24 (2): 481–488

6. D H BALABAN et al Low volume bowel preparation for colonoscopy: randomized, endoscopist-blinded trial of liquid sodium phosphate versus tablet sodium phosphate. Am J Gastroenterol 2003; Vol 98 (4): 827-832

7. E J LAI et al The Boston Bowel Preparation Scale: a valid and reliable instrument for colonoscopy-oriented research. Gastrointest Endoscopy 2009; Vol 69 (3): 620-625

8. A H CALDERWOOD et al Comprehensive validation of the Boston Bowel Preparation Scale. Gastrointest Endoscopy; Vol 72 (4): 686-692
