# Supplementary material for: A multicenter randomized phase 4 trial comparing sodium picosulphate plus magnesium citrate vs. polyethylene glycol plus ascorbic acid for bowel preparation before colonoscopy. The PRECOL trial
Source: Front Med (Lausanne). 2022 Dec 8;9:1013804. doi: 10.3389/fmed.2022.1013804 (PMC9773881; doi:10.3389/fmed.2022.1013804)
Supplement: Supplementary file 2 [file Data_Sheet_2.pdf]

OBJECTIVES OF THE STUDY

Primary objectives of the study

• To compare the efficacy, in terms of quality of bowel preparation, of the laxative based on sodium picosulfate and magnesium citrate (NapP) compared to the classic preparation with PEG, in patients undergoing diagnostic pancoloscopy.
• To compare the degree of acceptability of the bowel preparation with NapP compared to preparation with PEG.

Secondary objectives of the study

• to compare bowel preparation compliance with NapP versus PEG.

STUDY DESIGN

This is a multicentre phase IV, prospective, randomized, two-arm (1:1) study. The study will be conducted in open regarding the assignment of the treatment, as both the performer of the pancolonoscopy and the study subjects will be aware of the type of preparation assigned by randomization.
The assessment of the quality of the intestinal preparation will be carried out blindly, as this evaluation will take place through the analysis of the exam registration by two investigators who are different from the one who performed the exam, and who will not know the type of preparation assigned to that study subject.

ASSESSMENT OF THE QUALITY OF THE BOWEL PREPARATION

The assessment of the quality of the bowel preparation is the primary endpoint of the study. The evaluation will be blinded.

The quality of the bowel preparation will be evaluated according to the Boston Bowel Preparation Scale (BBPS). This scale provides a score between 0 and 3:

- 0: mucosa not visible due to the presence of solid stools that cannot be removed
- 1: mucosa only partially visible due to the presence of solid and / or liquid stools
- 2: mucosa visible despite the presence of minimal faecal residues, which can be aspirated
- 3: mucosa clearly visible, without faecal residues

The quality assessment according to that scale will be carried out for the following segments:

- Right colon (cecum and ascending colon);
- Transverse colon (including hepatic flexure and splenic flexure)
- Left colon (descending colon, sigma, rectum)

The global score can therefore be between 0 and 9.

Bowel preparation success is defined as a global score between 6 and 9, with a score of no less than 2 in every single segment.
Examples:

- Right colon 2, transverse colon 2, left colon 2: global score 6, success (satisfactory score in all segments).
- Right colon 1, transverse colon 3, left colon 2: global score 6, failure (unsatisfactory score for right colon)
- Right colon 1, transverse colon 3, left colon 3: global score 7, failure (unsatisfactory score for right colon)

EVALUATION OF THE ACCEPTABILITY OF THE BOWEL PREPARATION

At the time of the enrolment of the subject in the study, after having provided the subject the information relating to the study and have obtained the informed consent, a diary to fill out will be provided to the patient, together with the preparation and related instructions.
On the day of the exam, the patient will provide the informed consent to the execution of the pancolonoscopy and, before undergoing the exam, he/she will deliver the diary he/she had completed during the bowel preparation to the nurse and / or doctor with the aim of assessing its compliance with the solution used.
The diary completed by the patient will cover the following information:

- percentage of dose taken (100%, 75% or less)
- compliance with the timing of the assumption
- diet (VAS from 0 - excellent - to 10 - very bad)
- taste (VAS from 0 - excellent - to 10 - very bad)
- simplicity (VAS from 0 - excellent - to 10 - very bad)
- effects on personal activity (VAS from 0 - none - to 10 – activity impossible)
- effects on work activity (VAS from 0 - none - to 10 – activity impossible)
- availability to repeat the same preparation in a possible future colonoscopy
- general perception (VAS from 0 - excellent - to 10 - very bad)
- previous colonoscopies (yes / no; date of the last colonoscopy)

SAMPLE SIZE

The study is designed with the aim of recognizing a difference of 10% (considered as a minimum clinically relevant value) in the proportion of successes (adequate bowel preparation) between the two methods of preparation that are compared (primary endpoint).
Considering a success rate in the lower arm of 80%, a bilateral alpha error equal to 0.05, the study will guarantee 90% power in recognizing the expected difference of 10%, with the enrolment of 525 patients.

STATISTIC ANALYSIS

Compliance will be accurately described and compared between the two arms. The efficacy comparison analysis will be conducted on the basis of the intention-to-treat. The statistical comparison will be made with the chi-square test. In the primary analysis, the cases in which the colonoscopy is interrupted for reasons other than intestinal cleaning will be considered failures.
A sensitivity analysis will be conducted excluding the cases in which the colonoscopy examination will be interrupted before the evaluation of the 3 foreseen segments for reasons other than intestinal cleaning (i.e. stenosing lesions).
