# Supplementary material for: A multicenter randomized phase 4 trial comparing sodium picosulphate plus magnesium citrate vs. polyethylene glycol plus ascorbic acid for bowel preparation before colonoscopy. The PRECOL trial
Source: Front Med (Lausanne). 2022 Dec 8;9:1013804. doi: 10.3389/fmed.2022.1013804 (PMC9773881; doi:10.3389/fmed.2022.1013804)
Supplement: Supplementary file 3 [file Data_Sheet_3.PDF]

MODULO DI COMUNICAZIONE AL RICHIEDENTE DELLA DECISIONE DEL COMITATO ETICO RELATIVA AL PARERE UNICO[1]

Il presente parere del comitato etico è stato stampato dal sito internet dell'OsSC:

https://oss-sper-clin.agenziafarmaco.it

Da completare a cura del comitato etico che ha rilasciato il parere unico:

A. IDENTIFICAZIONE DELLA SPERIMENTAZIONE

A.1 Numero EudraCT : *2011-000587-10*

A.2 Titolo completo della sperimentazione: *SODIO PICOSOLFATO E MAGNESIO CITRATO VERSO POLYETHYLENE GLYCOLE (PEG) NELLA PREPARAZIONE INTESTINALE PER LA COLONSCOPIA: STUDIO RANDOMIZZATO DI FASE IV*

A.3 Codice,versione e data del protocollo del promotore:

Codice: *PRECOL2011-1*
Versione: *0*
Data: *03-02-2011*

Note:

1- Da trasmettere a cura del comitato etico entro trenta giorni dalla data di ricevimento della domanda nella forma prescritta (entro sessanta giorni in caso di sperimentazione monocentrica).

B IDENTIFICAZIONE DEL COMITATO ETICO<br>(costituito ai sensi del DM 12 maggio 2006)
**B.1 Denominazione del CE :** *COMITATO ETICO DELL`IRCCS ISTITUTO NAZIONALE PER LO STUDIO E LA CURA DEI TUMORI FONDAZIONE GIOVANNI PASCALE DI NAPOLI*<br><br>**B.2 Nome e cognome del Presidente:** *Prof. Francesco Paolo Casavola*<br><br>**B.3 Indirizzo del CE:** *VIA MARIANO SEMMOLA 80131 NAPOLI (NA)*<br><br>**B.4 Numero di telefono:** *0815903397*<br><br>**B.5 Numero di fax:** *0815903777*<br><br>**B.6 E-mail:** *comitatoetico@istitutotumori.na.it*

C. IDENTIFICAZIONE DELLO SPERIMENTATORE COORDINATORE (SE MONOCENTRICA, DELLO SPERIMENTATORE PRINCIPALE)
**C.1 Nome:**<br>**C.2 Cognome:**<br>**C.3 Centro clinico:**<br>**C.4 Indirizzo del centro clinico:** *VIA MARIANO SEMMOLA - 80131 - NAPOLI - NA*<br>**C.5 Reparto:**

D. DOCUMENTAZIONE ESAMINATA	
**D.1 Data della lettera di trasmissione della domanda:** *07/02/2011*	
**D.2 Data di ricezione della domanda:** *15/02/2011*	
**D.3 Data di ricezione di informazioni integrative:**	
**D.4 Modulo di domanda** *(CTA form)*	◉
**D.5 Documentazione riportata nella lista di controllo Ia del modulo di domanda**	◉
**D.6 Fascicolo della sperimentazione nell'Osservatorio in accordo alle informazioni del modulo di domanda**	◉

E. ELEMENTI VALUTATI<br>*(selezionare n.a. nei casi in cui l'informazione non sia applicabile)*		
**E.1 Dati di qualità del medicinale sperimentale(IMP/PeIMP)**		
Le informazioni e i dati necessari a supportare la qualità dell'IMP sono adeguati	◉	
Il promotore ha documentato che i prodotti in sperimentazione saranno preparati, gestiti e conservati nel rispetto delle Norme di Buona Fabbricazione (GMP) applicabili	◉	
E.1.1 Eventuali elementi critici riscontrati *testo libero)*:		
**E.2 Dati di farmacologia non clinica e tossicologia**		
Esistono presupposti solidi e rilevanti che giustificano l'avvio dello studio	◉	
E.2.1 Eventuali elementi critici riscontrati *(testo libero)*:		
**E.3 Dati clinici**		
Esistono presupposti solidi e rilevanti che giustificano l'avvio dello studio (non applicabile per studi di fase I e II)	◉	O N.A.
Lo studio consentirà di acquisire maggiori informazioni sull'IMP, di migliorare le procedure profilattiche, diagnostiche e terapeutiche o la comprensione dell'eziologia e della patogenesi delle malattie	◉	
E.3.1 Eventuali elementi critici riscontrati *(testo libero)*:		

E.4 Protocollo

Gli obiettivi sono coerenti con il razionale scientifico	◉	
Il disegno dello studio è pertinente e rilevante	◉	

<u>*Sono stati esaminati i seguenti aspetti*</u>:

Mancanza del gruppo di controllo	○	◉ N.A.
Disegno in aperto	◉	○ N.A.
Assenza di randomizzazione	○	◉ N.A.
Uso del placebo quale gruppo di controllo	○	◉ N.A.
Disegno di equivalenza o di non inferiorità	○	◉ N.A.
Lo schema di trattamento con l'IMP risulta adeguato (via di somministrazione, dosaggio e posologia, durata della terapia)	◉	
Il trattamento di controllo e lo schema di trattamento sono giustificati	◉	○ N.A.
I criteri di inclusione/esclusione sono appropriati, chiari e ben definiti	◉	
Gli esami, le visite e le procedure previste (specie se invasive) sono idonei a verificare gli effetti del trattamento	◉	
La misura di esito primaria è clinicamente rilevante o correlabile a una misura clinicamente rilevante	◉	○ N.A.
I metodi per rilevare la misura di esito primaria risultano adeguati	◉	○ N.A.
Il calendario previsto per la rilevazione dei parametri di efficacia è appropriato	◉	○ N.A.
I parametri selezionati per la valutazione della sicurezza sono congrui	◉	
Il follow-up ha una durata sufficiente in relazione all'obiettivo dello studio	◉	
La dimensione campionaria è stata calcolata in funzione della misura di esito primaria dichiarata	◉	○ N.A.
Il calcolo della dimensione campionaria è corretto in relazione alla potenza prevista per lo studio	◉	○ N.A.
Il piano statistico di analisi dei dati è coerente rispetto agli obiettivi	◉	
La differenza attesa tra i trattamenti confrontati è significativa	◉	○ N.A.
In caso di studio di equivalenza o di non inferiorità, la differenza considerata non rilevante è sufficientemente ristretta ed accettabile	○	◉ N.A.
Il protocollo è conforme alle linee guida EMEA in materia	◉	○ N.A.

Se sì al punto precedente, specificarne i riferimenti *(testo libero)*: *Linee Guida EMEA - Oncologia*

E.4.1 Eventuali elementi critici riscontrati *(testo libero)*:

**E.5 Aspetti etici**		
Il promotore ha documentato che la sperimentazione verrà condotta in conformità ai principi etici che traggono la loro origine dalla Dichiarazione di Helsinki, e che rispetta le GCP e le disposizione normative applicabili	◉	
I rischi e gli inconvenienti prevedibili sono stati soppesati rispetto al vantaggio per il soggetto incluso nella sperimentazione e per altri pazienti attuali e futuri	◉	
Il comitato etico è giunto alla conclusione che i benefici previsti dalla sperimentazione, terapeutici e in materia di sanità pubblica, ne giustifichino i rischi	◉	
I diritti, la sicurezza e il benessere dei soggetti dello studio hanno costituito le considerazioni più importanti e sono prevalsi sugli interessi della scienza e della società	◉	
La ricerca su persone che non sono in grado di dare il loro consenso informato è giustificata	○	◉ N.A.
Sono attesi possibili benefici diretti per il soggetto	◉	○ N.A.
Sono attesi possibili benefici per la collettività	◉	○ N.A.

E.5.1 Eventuali elementi critici riscontrati *(testo libero)*:

**E.6 Informazione dei soggetti e procedure per il consenso informato**		
Le informazioni per il paziente sono complete e comprensibili	◉	
Le procedure previste dal protocollo sono indicate in modo esauriente	◉	
I disagi ed i rischi cui il paziente potrebbe essere esposto sono ben descritti	◉	
Le modalità di ottenimento del consenso sono ben esplicitate	◉	
Le modalità di coinvolgimento di volontari sani sono adeguate	○	◉ N.A.
Le misure adottate per la salvaguardia della privacy del soggetto e la tutela dei dati personali sono appropriate, in accordo alla normativa vigente	◉	
Le modalità di informazione al medico curante sono corrette e complete	◉	○ N.A.

E.6.1 Eventuali elementi critici riscontrati *(testo libero)*:

E.7 Aspetti economici e informazioni relative a strutture e personale

Sono stati adeguatamente valutati gli elementi della proposta di contratto tra il promotore e il centro clinico	◉	○ N.A.
La copertura assicurativa garantisce un'appropriata tutela dei partecipanti in accordo alla normativa vigente	◉	
Gli importi, le modalità di retribuzione o di compenso o di emolumenti di qualsiasi natura, previsti dall'amministrazione di competenza per gli sperimentatori, sono conformi alle norme vigenti, adeguati rispetto all'impegno richiesto e non tali da costituire elemento determinante per la conduzione della sperimentazione	○	◉ N.A.
È stata considerata la congruità dell'eventuale indennità per i volontari sani, che non deve essere tale da costituire elemento determinante per la partecipazione alla sperimentazione	○	◉ N.A.
È stata esaminata l'idoneità dello sperimentatore e dei suoi collaboratori	◉	
La struttura sanitaria dove si svolgerà lo studio è appropriata	◉	
È stato verificato che il promotore dichiara di garantire una corretta e rapida diffusione delle informazioni acquisite attraverso lo studio	◉	

E.7.1 Eventuali elementi critici riscontrati *(testo libero)*:

F. DECISIONE DEL COMITATO ETICO		
**F.1 Sospensione della decisione** (ove applicabile)	○	
F.1.1 Acquisizione di informazioni integrative	○	
F.1.2 Modifiche alla domanda di sperimentazione	○	
F.1.3 Specificare la motivazione per la sospensione della decisione *(testo libero)*:		
**F.2 Parere unico favorevole**	◉	
F.2.1 In caso di richiesta di parere su una sperimentazione non commerciale il CE ha accertato la sussistenza dei requisiti della normativa vigente	si ◉	no ○ N.A. ○
**F.3 Parere unico non favorevole**	○	
**F.4 Sperimentazione da condurre presso**		
F.4.1 Stessa struttura	◉	
F.4.2 Altra struttura	○	
**F.5 Numero di pazienti previsti nel centro :**	*525*	
**F.6 Contributo lordo previsto dal promotore** (per soggetto completato, ove applicabile) :	*Non applicabile,Non applicabile*	

G. ASPETTI PARTICOLARI DELLO STUDIO CONSIDERATI NEL RILASCIO DEL PARERE UNICO FAVOREVOLE *(testo libero)*

H. MOTIVAZIONI DEL PARERE UNICO NON FAVOREVOLE<br>*(si può selezionare più di un'opzione)*	
**H.1 Protocollo**	
H.1.1 Rilevanza della sperimentazione	O
H.1.2 Criteri di inclusione ed esclusione	O
H.1.3 Gruppo di controllo	O
**H.2 Informazione dei soggetti e procedure per il consenso informato**	
H.2.1 Procedure per il reclutamento	O
H.2.2 Foglio informativo, modulo per il consenso informato e procedure	O
H.2.3 Protezione dei dati personali e confidenzialità	O
**H.3 Aspetti etici**	
H.3.1 Valutazione dei benefici e dei rischi prevedibili	O
H.3.2 Misure per minimizzare il dolore, il disagio e la paura	O
H.3.3 Inclusione di persone incapaci di dare validamente il proprio consenso informato e altre popolazioni vulnerabili	O
H.3.4 Adesione alle norme di Buona Pratica Clinica	O
**H.4 Strutture, personale e aspetti economico-amministrativi**	
H.4.1 Idoneità dello sperimentatore e dei suoi collaboratori	O
H.4.2 Adeguatezza della struttura sanitaria	O
H.4.3 Contratto tra promotore e centro clinico	O
H.4.4 Polizza assicurativa	O
H.4.5 Indennità per i partecipanti allo studio	O
H.4.6 Indennità per gli sperimentatori	O
H.4.7 Adempimenti degli obblighi amministrativi	O
**H.5 Altro**	O
H.5.1 Se si al punto precedente, specificare:	

I. DESCRIZIONE DELLE MOTIVAZIONI DEL PARERE UNICO NON FAVOREVOLE *(testo libero)*

L. SEDUTA DEL COMITATO ETICO

L.1 Data della seduta: *23/03/2011*

L.2 Numero del registro dei pareri del CE: *8-11*

L.3 Componenti del CE presenti e qualifiche:
PALMIERI LUIGI Medico legale
VECCHIONE ALDO Direttore scientifico (ex officio o suo sostituto permanente) dell'istituzione sede della sperimentazione
DANIELE BRUNO Clinico
RENDA ANDREA Clinico
ROCA FRANCESCO Esperto di bioetica
NIGRO RUGGIERO Clinico
DE SIMONE DOMENICO Rappresentante del volontariato per l'assistenza e/o associazionismo di tutela dei pazienti
IVALDO MARCO Esperto di bioetica
CASTALDO ANGELO Medico di medicina generale territoriale
FEBBRARO ANTONIO Clinico
LODATO SERGIO Direttore sanitario (ex officio o suo sostituto permanente)
CASAVOLA FRANCESCO PAOLO Esperto in materia giuridica e assicurativa
MAIOLINO PIERA Farmacista (ex officio o suo sostituto permanente) del Servizio Farmaceutico dell'istituzione di ricovero o territoriale, sede della sperimentazione

L.3.1 Sostituti permanenti che hanno partecipato alla seduta in vece dei membri *ex officio*:

L.4 Consulenti esterni presenti e qualifiche *(ove applicabile)*:

L.5 Componenti del CE presenti che non hanno partecipato alla votazione*(ove applicabile)*:

M. INVIO TELEMATICO DEL PARERE UNICO (comunicazione della decisione relativa al parere unico ai comitati etici collaboratori, per studi multicentrici)

M.1 Data: *25/03/2011*

N. FIRMA DEL PRESIDENTE DEL COMITATO ETICO *(compilare a mano)*

N.1 Il comitato etico ha espresso il parere unico:

- verificata la sussistenza del numero legale, essendo presenti membri n. *13* su n. *19*
- tenuto conto di eventuali osservazioni ricevute dai comitati etici collaboratori entro il ______________________

Si allega al presente parere l'elenco dei documenti esaminati (lista di controllo Ia) fornito dal richiedente con il modulo di domanda (CTA form).

29 MAR. 2011

N.2 Nome e Cognome: *Prof. Francesco Paolo Casavola*

N.3 Data: ______________________

N.4 Firma: Francesco Paolo Casavola

ISTITUTO NAZIONALE PER LO STUDIO E LA CURA DEI TUMORI FONDAZIONE G. PASCALE
