# Supplementary figures and images for: A multicenter randomized phase 4 trial comparing sodium picosulphate plus magnesium citrate vs. polyethylene glycol plus ascorbic acid for bowel preparation before colonoscopy. The PRECOL trial
Source: Front Med (Lausanne). 2022 Dec 8;9:1013804. doi: 10.3389/fmed.2022.1013804 (PMC9773881; doi:10.3389/fmed.2022.1013804)

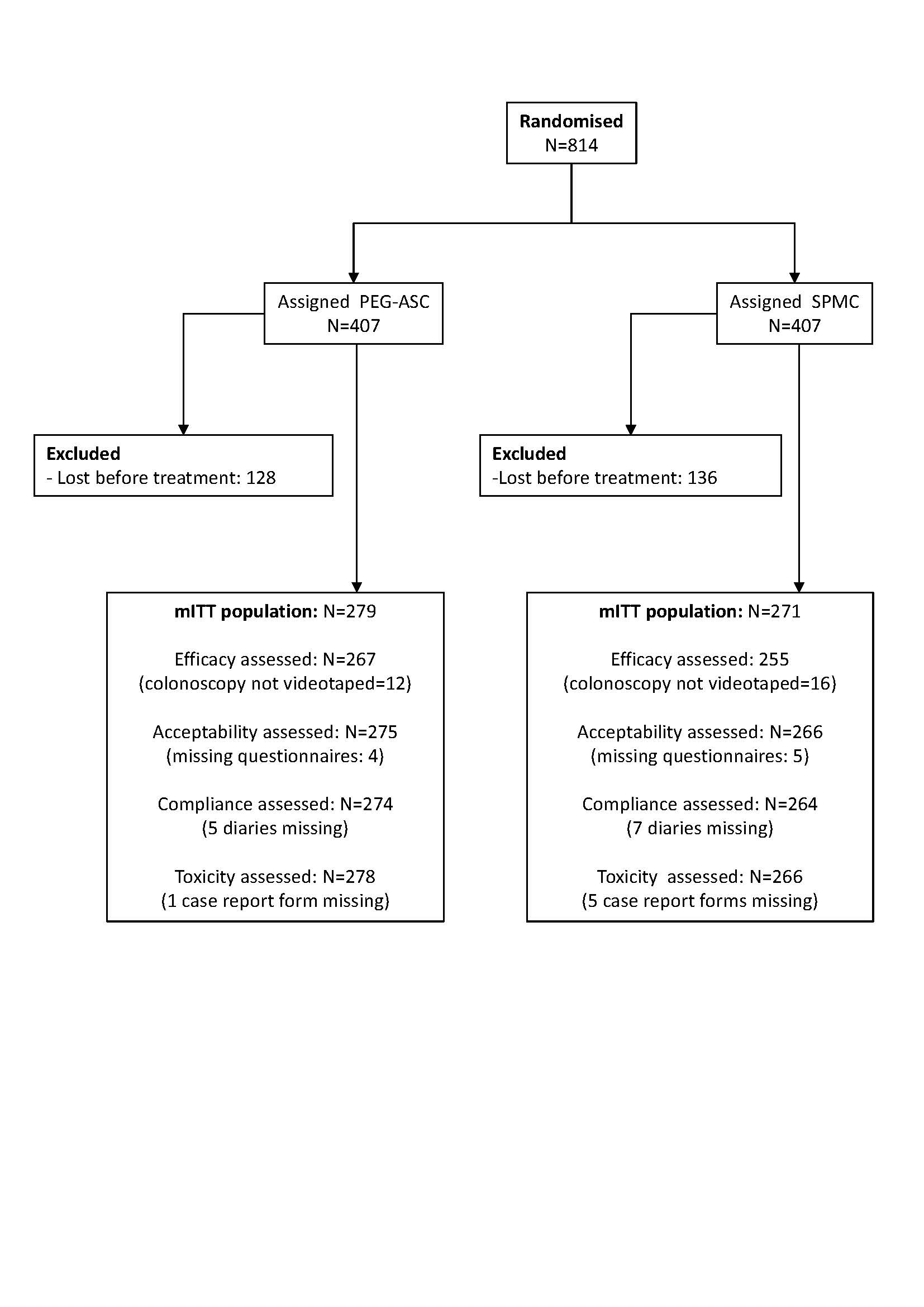

Supplement: Supplementary file 4 [file Image_1.jpg]
